# Impact of Imatinib Therapy on Immunometabolism to Promote β‐Cell Survival in Diabetes

**DOI:** 10.1111/1753-0407.70252

**Published:** 2026-06-30

**Authors:** Sandeep Kumar, Reena Kumari

**Affiliations:** ^1^ Tulane University School of Medicine New Orleans Louisiana USA

## 1 Introduction

Diabetes mellitus is characterized by progressive pancreatic β‐cell dysfunction, chronic low‐grade inflammation, oxidative stress, mitochondrial impairment, and systemic metabolic dysregulation [1, 2, 3]. Although currently available therapies effectively improve glycemic control, they fail to adequately preserve endogenous β‐cell mass and prevent long‐term disease progression [4]. Increasing evidence suggests that targeting immune metabolic signaling pathways may provide disease‐modifying therapeutic benefits by simultaneously modulating inflammatory responses and cellular metabolic homeostasis [5, 6].

Tyrosine kinase inhibitor imatinib has emerged as a promising therapeutic target for anti‐leukemic activity [7]. Beyond its direct effects on pancreatic β‐cells, imatinib also exerts broad immune metabolic actions that target several key pathogenic mechanisms underlying diabetes [8]. Studies have demonstrated that imatinib improves peripheral insulin sensitivity, suppresses pro‐inflammatory cytokine production, and attenuates chronic inflammatory signaling pathways that contribute to insulin resistance and β‐cell dysfunction [9, 10]. Inhibition of c‐Abl, PDGFR, and related tyrosine kinases reduces inflammatory stress, fibrosis, and tissue remodeling in metabolic organs, thereby improving systemic glucose homeostasis [11, 12].

Recent evidence indicates that imatinib induces a mild inhibition of mitochondrial respiration, leading to activation of AMP‐activated protein kinase (AMPK), a master regulator of metabolic homeostasis. AMPK activation subsequently suppresses thioredoxin‐interacting protein (TXNIP), reduces oxidative and endoplasmic reticulum stress, enhances autophagic responses, and protects β‐cells from apoptosis. In both experimental models and human islets, these effects preserve β‐cell viability and functional capacity under diabetogenic conditions [13].

## 2 Major Pleiotropic Actions of Imatinib in Diabetes

**FIGURE 1:**
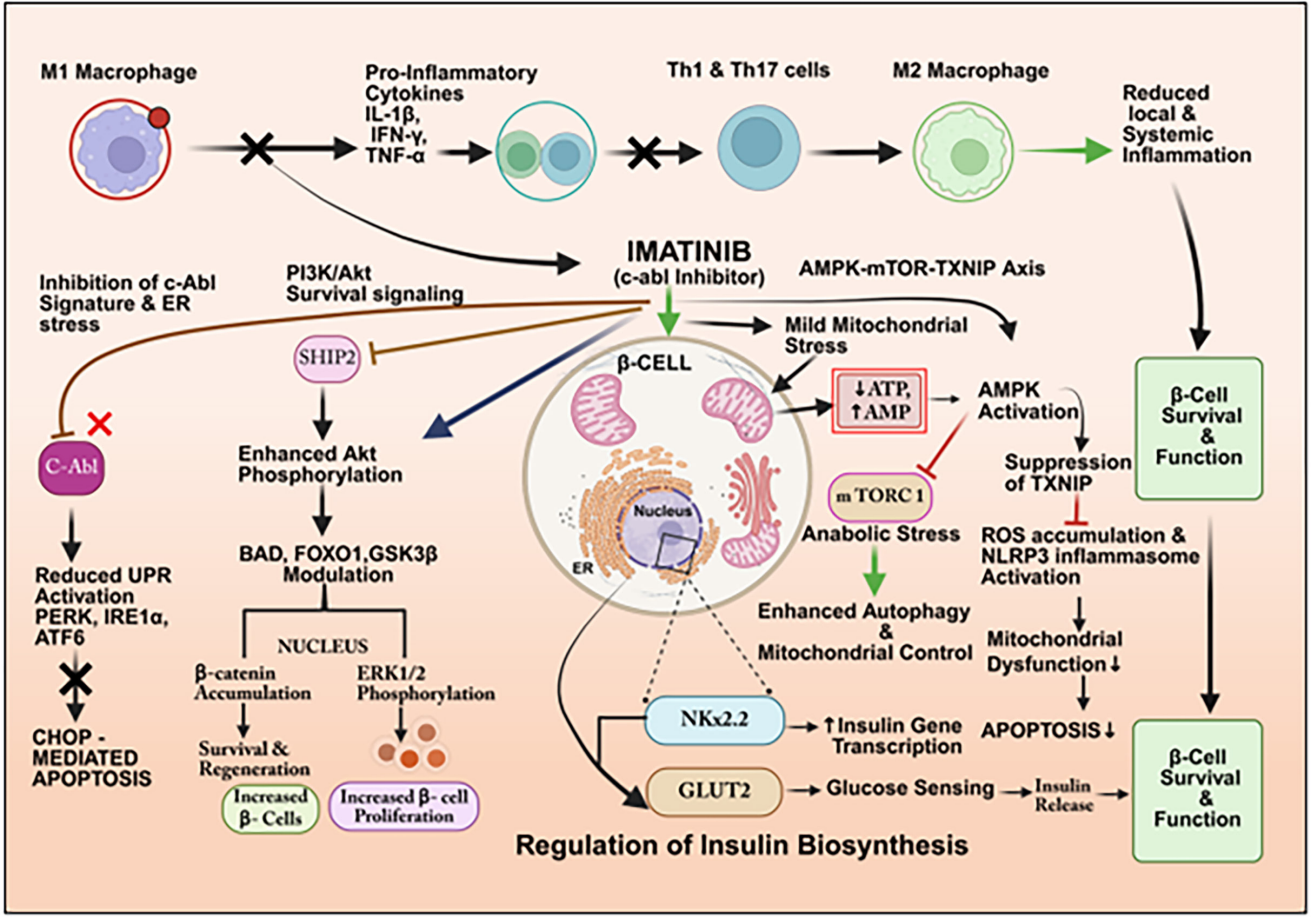
Proposed immune metabolic mechanisms of imatinib‐mediated β‐cell protection in diabetes. Imatinib inhibits c‐Abl signaling, attenuates ER stress and inflammation, activates PI3K/Akt and AMPK pathways, suppresses mTOR/TXNIP signaling, enhances autophagy and mitochondrial function, and promotes insulin biosynthesis, collectively improving β‐cell survival and function.

Imatinib exerts pleiotropic protective effects on pancreatic β‐cells through coordinated regulation of inflammatory, stress‐response, and metabolic signaling pathways [14, 15]. Imatinib suppresses M1 macrophage polarization, pro‐inflammatory cytokine production (TNF‐α, IFN‐γ, and IL‐1β), and Th1/Th17‐mediated immune responses while promoting an anti‐inflammatory M2 macrophage phenotype, thereby reducing local and systemic inflammation and reduce β‐cell death [16, 17]. At the cellular level, inhibition of c‐Abl signaling attenuates endoplasmic reticulum (ER) stress and suppresses PERK, IRE1α, and ATF6 activation, preventing CHOP‐mediated apoptosis. Concurrently, imatinib disrupts the c‐Abl–SHIP2 interaction, enhancing PI3K/Akt signaling, β‐catenin accumulation, and ERK1/2 activation, which collectively support β‐cell survival, proliferation, and regeneration [18, 19, 20]. Imatinib also induces mild mitochondrial stress, resulting in reduced ATP/AMP ratio and activation of AMP‐activated protein kinase (AMPK). Activated AMPK suppresses mTORC1 signaling and TXNIP expression [21], promotes autophagy and mitochondrial quality control, reduces reactive oxygen species (ROS) accumulation and NLRP3 inflammasome activation, and limits mitochondrial dysfunction [13, 22, 23]. Furthermore, imatinib enhances insulin biosynthesis and glucose sensing through upregulation of NKx2.2 and GLUT2 expression [24]. Collectively, these immune metabolic effects preserve β‐cell mass, maintain insulin secretory capacity, and improve glucose homeostasis in diabetes as shown in Figure 1.

## 3 Concluding Remarks & Editorial Take‐Home Message

Imatinib has shown pleiotropic actions by targeting inflammatory signaling, mitochondrial dysfunction, endoplasmic reticulum stress, and β‐cell survival pathways. Through coordinated regulation of c‐Abl, AMPK/mTOR/TXNIP, PI3K/Akt, and immune‐mediated mechanisms, imatinib represents a compelling example of how therapeutic repurposing can uncover novel disease‐modifying strategies. While further clinical validation is required, the success of imatinib highlights the potential of immune metabolic interventions to preserve endogenous β‐cell mass, delay diabetes progression, and redefine future precision therapies for diabetes.

## Author Contributions

S.K. and R.K. contributed to conceptualization, original writing and editing of the manuscript, and approved the final version of the manuscript.

## Funding

The authors have nothing to report.

## Conflicts of Interest

The authors declare no conflicts of interest.

## Data Availability

The data that support the findings of this study are available on request from the corresponding author. The data are not publicly available due to privacy or ethical restrictions.
