# Correction to “Impact of Imatinib Therapy on Immunometabolism to Promote β‐Cell Survival in Diabetes”

**DOI:** 10.1111/1753-0407.70294

**Published:** 2026-10-29

S. Kumar and R. Kumari , “Impact of Imatinib Therapy on Immunometabolism to Promote β‐Cell Survival in Diabetes,” Journal of Diabetes 18, no. 7 (2026): e70252.10.1111/1753-0407.70252PMC1331644942376693

The affiliation of the corresponding author was incorrect in the original publication. The correct affiliation is: “Independent Researcher, New Orleans, Louisiana, USA” instead of “Department of Microbiology/Immunology, Tulane University, New Orleans, LA, United States.”

We apologize for this error.

